# The Study of Labor Interests of Young Workers in the Selection and Adaptation of Personnel

**DOI:** 10.3390/bs10010022

**Published:** 2019-12-31

**Authors:** Tatiana Lobanova

**Affiliations:** Department of Human Resources Management, National Research University Higher School of Economics, 101000 Moscow, Russia; lobanova@hse.ru; Tel.: +7-903-728-9617

**Keywords:** motivational mechanism, labor interests, personnel selection, adaptation, the structure of interests

## Abstract

The issue of actualization of labor interests as a motivational driver and one of the sources of labor productivity has received little study in applied and organizational psychology. The study given in the paper fills some “blanks” of this problem. The leading approach to research is the mechanism of motivation, including the phenomenon of interest in work proposed on the basis of theoretical analysis. Using the methods of a special survey, questioning, and interview guides, the analysis and comparative assessment of the labor interests of 50 candidates for the service manager position (entertainment and restaurant industry field) was conducted, as well as of 45 employees in this industry aged 18 to 25. The main results of the paper show the connection of labor interests with the company’s personnel management system, namely, with the processes of selection, adaptation, and training. It was revealed that the candidates selected for the service manager position were of primary group interest and financial incentive was secondary. At the same time, financial incentive contributed to the successful passage of the adaptation period. Occupational interests of employees depended on their education and job specifics. Career interests were influenced by the time spent with the company. The recommendations necessary for employers to create the conditions corresponding to the leading labor interests of employees were substantiated.

## 1. Introduction

Field practices of personnel management allow the company to build effective systems of screening and selection, adaptation, motivation, and training of employees. Particular attention is paid to two processes: the selection of personnel and its adaptation, as the acquisition of personnel is a key task for the system of human resources management. Often when business only starts to develop or there are restrictions in financial means, entrepreneurs look for interested candidates who will put their best at work and have high professional and team motivation. 

To provide the company with the “proper” staff, it is necessary to understand what labor interests and expectations employees already have, and what labor interests might be supported and developed by the organization itself. When the interests of employees are congruent with their work environment, and employees feel able to use their own competencies, then we have higher levels of involvement and inner motivation to work. As shown by research, all these factors have the best effect on the performance [1] (pp.73–90) [2] (pp.99–108). The area of intersection of interests of employees and interests supported by organization forms a request for the search for new employees, for their fastest and most effective introduction into a company and development of the internal potential according to the existing requirements [3] (pp.90–100) [4] (pp.2–17). 

Introduction of an additional parameter to hiring, such as labor interest, allows to assess the specifics of the employee’s personality and eventually to obtain an employee, in whose “fiery eyes” the manager will notice, regardless of the content of the job, interest in the work, 100% involvement, and maximum return. 

The logical continuation of the selection practice in the personnel management system is the adaptation and training of personnel. The statistics say that “about 80% of the people resigned within the first year of work actually decided to leave the company within the first two weeks” [5] (pp.200–207). The adaptation process for new employees requires financial and time costs, so it is not profitable for companies to hire those who are quick to resign. At the same time, it is more important to shorten the adaptation period and get the most out of the new employee.

The article presents the results of a pilot study of the candidates’ labor interests during the selection, adaptation, and training of personnel in one of the service companies of the entertainment and restaurant industry in Moscow (Russia) in 2017.

The purpose of the study is to show the importance of taking into account the workers’ labor interests and the importance of influencing them in the process of personnel management.

The article’s hypothesis is that in the process of selecting the optimal personnel for a service company, the labor interests of a candidate play an important role. Based on the identified labor interests of candidates, it is possible to ensure reliable selection and sustainable adaptation of personnel. If a company does not take into account the labor interests of employees and does not shift them towards a professional component and sustainability, it might result in poor employees’ adaption, and their eventual migration to those competitors who are more sensitive to satisfying these interests. Depending on the category of personnel and type of activity, priority labor interests differ.

The initial hypothesis contains a number of particular hypotheses (see below), which have been proved by constructing contingency tables.

Despite a significant body of academic literature relevant to the analysis of various aspects of economic, professional, public, and personal interests published in the second half of the 20th century, a number of insufficiently studied theoretical and practical questions still remain [6] (pp.185–191) [7] (pp.44–45) [8,9]. Among them are the historical, gnoseological, and ontological nature of labor interest, and the factors that determine it; forms and conditions for the realization of interests under modern circumstances; the role and place of labor interests in the economic system of society; regularities in the formation, functioning, and development of labor interests; and trends in their transformation. The problem of determination and dynamics of the phenomenon of such interest is also insufficiently developed in the area of social psychology, labor psychology, and organizational psychology [10]. According to Eberhard Todt [11], interests are relatively long and generalized tendencies of behavior, closely related to the development of the image of the ego. Karl Mierke singles out the phenomenological, functional, and structural-psychological aspects of the consideration of interest [12]. As stated by H. B. English & A. Ch. English, interest mattered as the importance of attitude or focuses of attention, that is, the sense of significance [13]. According to the Soviet psychologist and philosopher S.L. Rubinstein, interest in the psychological sense of the word is a specific orientation of the individual, consisting of the concentration of his thoughts on a particular subject [14]. E. P. Ilin Doctor of Psychology, Professor of St. Petersburg University carried out detailed theoretical analysis of the notion of interest in 2002 [15]. He classifies interest as “types of motivational formations”, including motivational states, motivational attitudes, drives, desires, volitions, inclinations, and habits. At the same time, interest appears a key component of the motive, determining personal preferences in the needs and ways of satisfying them, in the desire to act. James Rounds and Rong Su proposed a theoretical model that points to the relationship between interest, motivation, behavior, and result. The presence of interest activates the process of motivation, influencing the formation of activity goals, sustainability, and focus of efforts. This mechanism forms program–target behavior, leading to clear-cut motivational results. Accordingly, the more stable is the labor interest in the activities, the more efforts an employee exerts, resulting in higher effectiveness and performance [16] (pp.98–103). According to another theory by Michael Ingerick and Michael G. Rumsey [17] (pp.165–181), there are three key determinants of labor behavior: motivation, knowledge, and experience, and occupational skills (habits). Michael Armstrong refers motivation to the “factors that cause people to behave in a certain way” [18]. Before the labor action, explaining and justifying it, motivation can serve either as an impulse for action or as a means of blocking it. However, the motive itself is not a driving factor, but acts as a link between such regulators of labor behavior as needs, interests, goals and values, attitudes, and standards.

**Figure 1:**
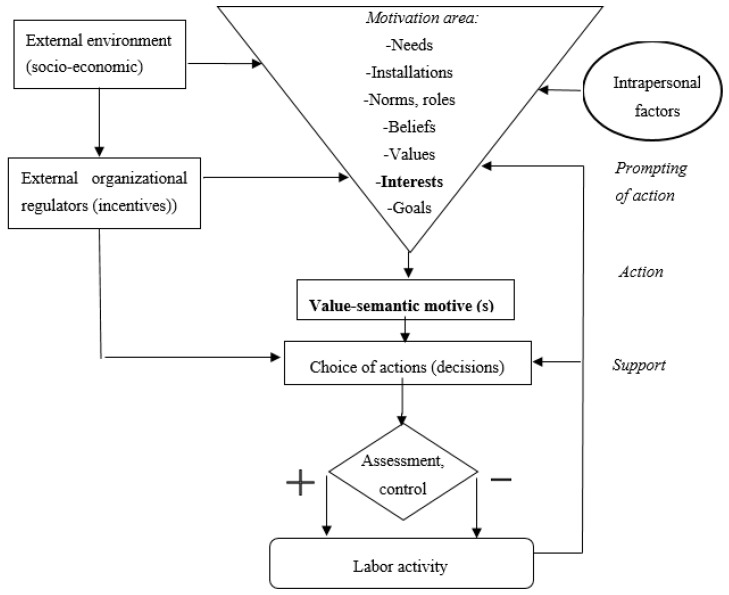
The mechanism of motivation for activity and place of interest.

The research conducted into the psychological aspects of labor motivation and interests has led to a proposition linking motivation mechanism to activity (Figure 1). This mechanism is influenced by external factors such as social reality and the working environment, and some internal factors [19] (pp.26–45). 

Needs “activate” the person or organization and launch the mechanism of labor motivation. However, only the conscious needs can create propulsion: When employees or company leaders, realizing that external conditions do not meet their internal requirements, carry out activities to eliminate such a discrepancy. Conscious needs acquire the form of interests to specific objects of labor that ensure the satisfaction of needs. On the dominant interest, in combination with the real status of a person, a system of orientation values are defined—a relatively stable, socially conditioned attitude to the aggregate of material, spiritual goods and ideals, on the basis of which the desire to achieve certain goals arises. “The modern motivational mechanism includes also the paradigm of employees’ attitudes—the state of the person’s psychological readiness to behave in a certain way in relation to the object, determined by past experience” [20].

Thus, certain structural elements of the motivational mechanism—needs, interests, values, attitudes—encourage, i.e., launch different motives through incentives for the performance of an action within the established norms, rules, and other aspects of the economic and social environment and labor situation. This triggers behavior, i.e., there is a motivation for a person to a certain labor action. Next is the implementation of the action and the person receives an assessment (reward or punishment) for the implementation of actions. Here, there is either a weakening, or preservation, or an intensification of action motivation. Monitoring and maintaining the behavior aimed at achieving the goal are expressed in certain persistence in achieving this goal. And the motivation itself makes a person interested. Thus, we see that when considering the modern mechanism of labor motivation, interest takes a key place, playing the role of a “lighting up” trigger, a motivational impulse giving a positive result. 

This mechanism forms a motivational and stimulating environment that allows to seriously affecting the labor behavior of employees to achieve effective results. Thus, we define labor interest (interest in work) as a value, emotionally colored position of the employee’s personality in relation to personal field of activity [21] (pp.95–106). Personal success of the employee, as well as the achievements of the enterprise organization, and even the economy as a whole may depend from the proper maturity of this position. If an effective personnel management system is initially built in the company—where the selection procedures will identify the true interests of candidates, and also interests of the company are taken into account; the system of adaptation and training will increase labor interest or adjust it depending on requirements; and the company can obtain highly motivated employees.

It is obvious that interests can grow and develop, but they can gradually weaken, and finally disappear altogether without taking care of their maintenance. Labor interest is stable, but it also can be influenced and modified. In different periods of a person’s life, in the process of gaming, training, imitation, design, adaptive, cognitive, and labor activities, interests develop and evolve. The number of practical studies in this area is insignificant, but they emphasize the interrelation between interest and work [22] (pp.42–52) [23] (pp.111–127). In this regard, the relevance of this study is to bring new arguments to prove the development of labor interests in the process of adaptation and work itself.

## 2. Materials and Methods

The methodological difficulties in the study of labor interests revealed a lack of available questionnaires of interests that refers more to the professional preferences (for example Strong Interest Inventory [24], Klimov Table for an approximate determination of the preferred type of prospective specialty [25]); interests of different social and professional groups (buyers, advertisers, realtors, youth, and women); or explored cognitive interests.

Psychologists, economists, sociologists, and legal professionals provide different classifications of interests. The problem with these classifications was due to the fact that they all considered the interests of a person beyond the context of his/her organizational behavior. 

The author’s methodology for assessing the labor interests of workers is based on motivation for activities in which interest plays a leading role. Interest in work can arise at first as an emotion, accompanied by attention to a specific piece of work and the desire to learn more about it. With a successful implementation of this aspiration, the interest acquires stability and structure.

Based on the cluster content analysis of in-depth interviews with employees of various commercial companies, the author has proposed a classification of labor interests (see Table 1). The selection of these seven clusters (types) is based on the personal components of their carriers—on the content of interests and targeted orientation of workers.

Such an ensemble of interests takes into account social and economic environment, and the organizational conditions in which the employee is situated, personal (incl. hereditary) characteristics, as well as self-determination and personal dynamics.

In the assembly of labor interests, one of the seven types listed in Table 1 begins to “prevail”, and others move to second or third place. Leading sustained interest, reinforced by success, brings up evaluation of actual employment as “meeting the needs”, and causes many positive emotions, i.e., actually causes satisfaction with work. Increasing positive emotions, in turn, leads to the desire to identify with work, i.e., to involvement in the labor process. In case of being dissatisfied within employment, interest becomes unstable, and it decreases. With a weak team spirit and an unfair salary, from the individual’s point of view, job satisfaction is reduced. If the valuation of the employee and the organization do not match, job satisfaction will not lead to involvement in the labor process. However, in the presence of favorable factors, interest in work gains stability and develops into involvement [26] (p. 86). In fact, involvement means integration into the company’s life, the willingness of the employee to make significant effort, the desire for self-fulfillment and high labor activity. Labor activity is expressed by various indicators, for example, quality of work carried out, labor productivity, use of new equipment, advanced methods and technologies, compliance with internal rules, regulations and technological discipline, and also employee’s involvement and participation in the improving of production and labor management [27] (pp. 151–158).

This model of labor interests was the methodological basis for developing a method for investigating labor interests, using the questionnaire entitled the Labor Interests Survey. It included a series of statements such as “My compensation corresponds to my level of professionalism”, “My employment activity is so fascinating that sometimes I forget about the end of the working day”, or “I’m proud of my company’s achievements.” It was necessary to select the answer reflecting the importance of each statement for the respondent: from totally insignificant (1 point) to very important (5 points). By the number of points scored, the priority of one or another type of interest was recorded. The methodology Labor Interests Survey was validated and tested for reliability and consistency.

The study was conducted on the basis of a service company, which is one of the leading players in the restaurant and entertainment field which consists of a management company and 12 branches. In total, the company employs more than 1200 people. The main problems of human resources management in the company were concentrated on the grounds of service personnel selection, adaptation of employees, and their retention within the first year. The staff was randomly selected and had a high turnover. The task was to optimally select and adapt staff. Therefore, it was important to understand which of the candidates had the highest and lasting interest in the job.

The study was conducted in several stages: a pilot study, then a modification of the initial questionnaire and collection of information for further analysis. Within the framework of the pilot survey (questionnaire), 45 employees working in the company were interviewed. As a result of the analysis, additional questions were added to the main elements of the Labor Interests Survey. Also, the scale was changed from ordinal to nominal. Developed according to the author’s technique Labor Interests Survey it has been modified for two groups: for the candidates applying for the position of service manager and for employees on a probationary period. In the course of the survey, the candidate’s expectations were clarified, and special attention was given to identification of the main labor interests and their hierarchy, hobbies, and their involvement in various spheres of life. Thus, a preliminary pilot study allowed us to clarify the use of various research incentives not as random factors, but to focus on the types of labor interests.

The questioning of the candidates was conducted before the initial interview in order to obtain the most independent, valid, and reliable results. The questioning was repeated a month later with the company’s employed candidates to assess the effect of the adaptation period. 

For further quantitative analysis, 50 questionnaires filled out by the candidates were selected: 25 were filled out by candidates who successfully passed the interview and subsequently found a job with the company; another 25 were filled out by candidates who did not pass the selection stages. The age of respondents was from 18 to 25, but all candidates had to work full-time. Men accounted for 52% of the total number of respondents; women—48%. The candidates interviewed had secondary vocational or incomplete higher education degree. This sample was limited by the number of applications from candidates, and a period of time allocated for selection. An increase in sample’s size would mean incorrectness and incomparability of these candidates would have to consider previous year’s candidates when there were different terms of employment. Note that the individual characteristics of candidates were distinguished by sufficient diversity and inclusiveness. Although they all lived in Moscow, they represented different regions of Russia: the Far East, Siberia, the Urals, the South, the Volga Region, Center, and the Northwest. Representatives of other countries of the near abroad (Belarus, Ukraine, Kazakhstan, and Kyrgyzstan) were also included in the sample. The nationalities of the candidates also varied: apart from Russians, there were Tatars, Yakuts, Karelians, Bashkirs, Belarusians, Ukrainians, Kazakhs, Kyrgyz, and others. Common to all candidates were age characteristics, secondary education, and a desire to work in a company in this business sector.

It is known that when using questionnaires, the individual’s answers to direct questions about his interests are often the most unreliable method of such interests’ identification and evaluation. Therefore, to confirm or disprove the quantitative results identified by the questionnaire method, qualitative methods were used: analysis and evaluation of applicant documents (CV of those candidates who helped assess the trajectory of interests development), interviewing, which was conducted in the format of an interview guide, and allowed more detailed understanding of a candidate’s previous experience and the orientation and sustainability of labor interests; professional skills test, helping to assess the priority of occupational and career work interest.

Next, a qualitative study was conducted to verify the quantitative research. It was conducted in the form of a guide interview about the interests of candidates. Only 28 candidates (14 men and 14 women) out of original 50 participants agreed to take part in such interviews. The respondents’ answers were divided into several categories: the relationship of interests with HR-functions, the labor interests of employees, the perception of labor interest, values, etc. These interviews were used as the basis for quantitative research. As a result, the same priority clusters (types) of interests were identified as in the quantitative study.

In accordance with the personal data protection policy, the results of a quantitative and qualitative analysis of the labor interests of candidates were provided to the service company in a generalized form.

## 3. Results

### 3.1. The Reasons for Choosing a Job in Company

In the course of the quantitative study, a frequency analysis of the responses was carried out along with the construction of the cross table, conjugacy tables, and the correlation matrix. Frequency analysis based on the results of the candidates questioning showed that categories of peoples’ interest in work are as follows.

- Friendly team environment—80%.
- Interesting and diverse content of actual labor—62%.
- Personal commitment—56%.
- The opportunity to get a good salary—52%.

**Figure 2:**
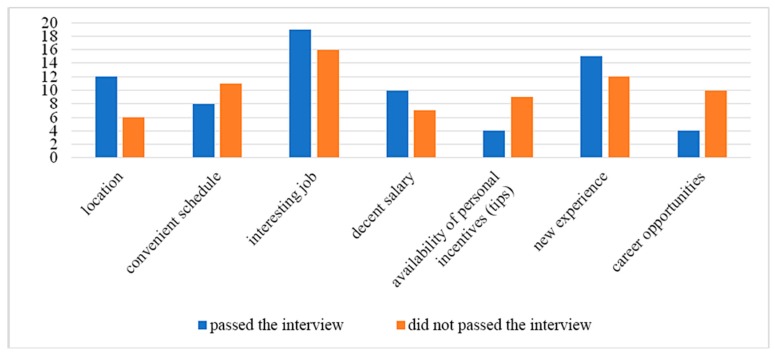
The summary of answers to the question: “Why did you decide to join our company?”.

Figure 2 contains the results of respondents’ answers about the reasons for choosing a job in a particular company. When answering this question, the interests of those who passed and did not pass the evaluation interview differed. 

### 3.2. Communication of Labor Interests with Personnel Management Processes

Quantitative analysis made it possible to collect statistical information, on the basis of which significant differences in labor interests between successful candidates admitted to the company and candidates who did not pass the selection stages, were revealed. 

An analysis of the cross-table captures the distribution of answers between what contributes to the interest in work and what matters most in ensuring a proper employee’s performance showing the following distribution of preferences among the interviewed. Working in a friendly team and trusting relationships stand in the first place (62%). Wages associated with proper performance and labor interest come second (46%). Finally, personal sense of purpose is essential for professional development (34%) and interesting and diverse content of actual labor matters (34%).

Within the framework of the initial hypothesis, five additional hypotheses were identified (see Table 2), and were verified by constructing conjugacy tables (The contingency coefficient (Cramér’s V) is a measure of the connection based on chi-square. This value changes between 0 and 1, where 0 means no relationship between the row and column variables, and a value close to 1 means a high degree of relationship between these variables.). The correlation analysis was carried out using the software SPSS Statistics, R. 1. The confirmed hypotheses were estimated according to the Cramer’s Coefficient. Values less than 0.15 reflect the presence of a weak bond and those between 0.15 and 0.3 show an average degree. Figures above 0.3 represent high degree of the connection between the indicators. 

Hypothesis 1.1 was confirmed with a maximum degree of probability (*p* = 0.000). Eighty percent of respondents believe that the friendly team facilitates interest in work. At the same time, 58% noted that they may perform an uninteresting task or the job if their team interests coincide with others (in non-formal communication). 

Hypothesis 2 on the availability of an open position in this company that offers attractive salary and payment system, including the presence of individual incentives (tips) was confirmed with an asymptotic significance of 0.009. Only 40% of respondents emphasized the importance of financial labor interests.

Candidates with less interest in a career development are more successfully interviewed for the positions of the line service personnel. Hypothesis 3.1 was confirmed with a probability degree of *p* = 0.059. Most interviews were successful and led to further employment for those candidates who did not emphasize the importance of career growth. Twenty-eight percent of all interviewed candidates noted the importance of career development in their work, but only 8% eventually found a job with the company. 

Candidates intending to work for the company for more than one year were interested in their career path progress. Hypothesis (3.2) was confirmed with a probability of *p* = 0.026. This means that those candidates who are counting on long-term cooperation with the company are determined to realize themselves in the future in order to achieve a bigger progress. 

Candidates who have higher (or incomplete higher) education consider the position at the service personnel level as temporary in contrast to candidates with secondary-level education. Hypothesis 3.3 was also confirmed (*p* = 0.026). Candidates with secondary-level education are more profitable for this employer as they are ready to work longer (more than 1 year). Also, professional development is most significant for those candidates whose education is at secondary level, as they plan to develop in the direction they have chosen (Hypothesis 4 was confirmed with *p* = 0.044). Finally, Hypothesis 5 completely confirmed that employees who previously had such entertainment employment experience assess more objectively their ability to quickly pass professional adaptation (*p* = 0.000). 

## 4. Discussion

An interesting fact was that corporate interests differed according to gender. Women demonstrated that their corporate interests, such as “job in entertainment”, “the stability of the company is significant” is higher (70%) than for men (50%).

It was revealed that there is a high degree of connection between professional and career interests (correlation coefficient r = 0.369). If the value of the variable “I like the work I plan to do, because I want to become a master of my craft” is increasing, then the value of the variable “I will achieve high results as the service manager to take a higher position” also increases.

In the process of the study, it was noted that in the first months of probation, the staff had a difficult time undergoing professional adaptation, while social adaptation occurred very quickly, often just within 1–2 shifts. Out of the 25 employed candidates, 20 (80%) stayed with the company after the probation. Three candidates resigned within the first week of employment. Two candidates were dismissed because they failed to pass the necessary professional tests after a month of employment. It turned out that the process of adaptation was positively influenced by the economic interest (the full-time employee salary), and the group interest (team/workforce) came second. The wage increases after the passing of professional tests motivated employees to quickly pass professional adaptation.

The answers to the question asking to explain the reasons for joining this particular company (of the respondents who passed and did not pass the evaluation interview) differed. The candidates who passed the interview pointed out “interesting job”, “new experience”, and “convenient location” reasons. These candidates were ready to adjust to the schedule and the existing system of payment, and most importantly, displayed a strong interest in this employment. Therefore, the conclusion was drawn that a candidate’s financial interest was more declarative than real.

Unexpectedly, it turned out that in this category of service personnel, primary group interests (interests of small groups) were more important than financial (economic) interests. Why did the interests of small groups (shifts, brigades) have a key impact on labor behavior and the work process? It is presumed that new employees gave preference to the friendly team environment because they were inexperienced and did not know all the professional and functional subtleties of the job. Such candidates counted on their team in their expectation of assistance during professional adaptation period.

Those candidates, who were dominated by financial labor interest, openly stated that they intend to resign in the near future since they want to earn more. However, the increase in wages after training and passing the test motivated employees to speed up the adaptation and stay longer with the company. 

A more detailed analysis of the data led to the conclusion that career labor interests were not a key consideration to the staff. The candidates who did not distinguish the importance of career growth had more success during the interviews and, as a result, were mostly employed by the company. However, the presence and extent of career interests depended on the planned time of candidate’s employment with the company. The longer the candidates planned to stay with the company, the more they were interested in developing their knowledge and skills. 

The results of the study confirm the findings of other authors, which were mentioned in the introduction. Thus, the strong degree of connection between consent to delayed salaries and the importance of trusting relationships in a team (see Table 2 hypothesis 1.1) is well combined with the conclusions of Johnson V.A. and Beehr T.A. [2] about high intrinsic motivation to work when the interests of employees are congruent with the work environment. A high degree of connection between work experience and the term of qualification training (see Table 2 hypothesis 5) allows hired candidates to quickly and efficiently enter the company and develop. On the whole, this corresponds to the discussions in the article by Vahidi N., Roslan S., and Chong A.M. [3]. In addition, the study presented in this article is consistent with the Rounds J. and Su R. [16] model on the strong influence of interest, which forms the program–targeted behavior.

## 5. Conclusions

Thus, the main hypothesis was confirmed—in the process of selecting the optimal personnel for the service company, high level of labor interests of a candidate are playing an important role. A thorough and comprehensive assessment of these interests helps to shorten the period of adaptation and minimize the turnover of personnel within the first year of work. There was a difference in labor interests among different categories of line staff. For example, in one category (customer service personnel), narrow-group interests prevailed. These employees worked for the sake of gaining experience in communication hoping to obtain more promising employment in the future. In the staff function category, the professional interest (obtaining new knowledge) was higher than other categories. 

In conclusion, due to different interests of workers, the process of their management must be diverse. In turn, the level of labor interests, including the entire assembly of interests listed in the article is based on individual perception, which is determined by internal motives, values, and attitudes. The employers, creating conditions that correspond to the interests of employees positively affect the latter’s perception of the employment with the company and significantly reduce the level of staff turnover. 

As a result of the conducted research, based on the example of the entertaining industry company personnel, we received evidence of the importance of taking into account the employees’ labor interests and positively influencing them in the process of personnel management. Reliance on the identified labor interests of candidates contributes to the effective selection and sustainable adaptation of personnel. We saw a difference in the structure of the interests of the line and functional staff and formulated the following recommendations for the management of the company:

1. If companies seek to retain professionals, then it is necessary to provide such employees with the opportunity to feel their own importance, to fully utilize their abilities by enriching the nature of assignments, and provide appropriate assistance and resources, including occupational training.

2. A system of corporate team building activities, including unifying rallies, meetings, corporate holidays, competitions, sports, travel, quests, etc., is very important for retention of employees with the predominance of group or corporate interests. Internal marketing systems and creation of “organizational memory”, “integrated space”, and “charity fundraising” are also becoming popular. This entire system works to reduce the risks of loss of human assets and avoidance of dependencies on “personalities”.

3. Top management should pay more attention to emerging conflicts and maintaining microclimate in small groups. At the same time, management should be able to change the remuneration system during the period of professional adaptation of employees.

The practical use of the results of this study for HR and line-managers is that the personnel procedure is simplified: if we see a candidate exhibiting a high level of interest for work in small group, then most likely it will be a successful and highly motivated employee. She or he will work for a long time in the company. For line managers, an important practical result of the study is that during the adaptation period it is necessary to take into account the economic interests of employees and pay attention to the need for financial support.

## Figures and Tables

**Table 1:** Types and content of labor interests.

Interest Groups	Types of Labor Interests	Content of Interests
Individual interests	1. Economic interests	The employee is interested in the amount of salary and its components, ways to increase, or other financial benefits
	2. Occupational interests	The employee is interested in self-development within the position held, and also in improving professional skills and gaining proficiency.
	3. Career interests	Interest in further advancement and opportunity for career growth.
Group interests	4. Primary group interests	The employee is interested in the team specific aspects and emotional climate, paying attention to the manager-subordinate relationship.
	5. Corporate interests	Interest in corporate values, corporate culture, events, attention to the specifics of the company’s performance and results.
Public interests	6. Community (local and/or national) interests	The employee performance is subordinated to local or national interests, customs, community practices or traditions, worldview values.
	7. General civil interests	The employee is focused on serving people, citizens of his country, the state and society, committed to patriotism and fulfillment of civil duties

**Table 2:** Summary table reflecting the level of communication stability.

Hypotheses		Cramér’s V	Indicators
1. Line service personnel are primarily focused on the interests of small groups; the team/workforce is of significant importance, as well as its internal relationships	Hypothesis 1.1	0.659	A strong degree of connection between consent of salary delays and the importance of trust within the team
	Hypothesis 1.2	0.488	A strong degree of connection between consent of salary delays and the significance of being part of the team
2. Financial labor interests are significant in the Service personnel performance	Hypothesis 2.	0.392	A medium (average?) degree of connection between the choice of employment and wages
3. The line staff is not concerned about career labor interests	Hypothesis 3.1	0.267	A medium degree of connection between career interests and choice of employment with this particular company
	Hypothesis 3.2	0.379	A medium degree of connection between prospective period of employment with the company and career interests
	Hypothesis 3.3	0.332	A medium degree of connection between education and career interests
4. Professional development at work is of most significant importance for candidates with secondary-level education as they intend to evolve in the chosen direction	Hypothesis 4	0.323	A medium degree of connection between education and professional interests
5. Candidates with extensive work experience adapt and learn faster	Hypothesis 5	0.541	A strong degree of connection between work experience and the terms of qualification training.
